# Supplementary material for: SLAMF6 compartmentalization enhances T cell functions
Source: Life Sci Alliance. 2022 Dec 8;6(2):e202201533. doi: 10.26508/lsa.202201533 (PMC9733572; doi:10.26508/lsa.202201533)
Supplement: Supplementary file 1 [file LSA-2022-01533_TableS1.docx]

Supplementary Table

OKT3-IgG-hole

ATGGGCTGGAGCTGCATTATCCTGTTCCTGGTGGCCACAGCCACCGGCGTGCACAGCCAGGTGCAGCTGGTGCAGAGCGGAGCAGAGGTGAAGAAGCCAGGAGCCTCTGTGAAGGTGAGCTGCAAGGCCTCCGGCTACACCTTCACACGGTATACCATGCACTGGGTGAGACAGGCACCTGGACAGGGCCTGGAGTGGATGGGCTACATCAACCCAAGCCGGGGCTACACAAACTATAATCAGAAGTTTAAGGACAGAGTGACCATCACAGCCGATAAGAGCACCTCCACAGCCTATATGGAGCTGAGCTCCCTGAGGTCCGAGGACACCGCCGTGTACTATTGCGCCCGCTACTATGACGATCACTACTGTCTGGATTATTGGGGCCAGGGCACCACAGTGACAGTGTCTAGCGGCGGAGGAGGCTCTGGAGGAGGAGGCAGCGGCGGCGGCGGCTCTGGCAGCGAGATCGTGCTGACCCAGTCCCCAGCCACACTGTCCCTGTCTCCAGGAGAGAGGGCCACCCTGAGCTGCTCCGCCTCCTCTAGCGTGTCTTACATGAATTGGTATCAGCAGAAGCCCGGCAAGGCCCCTAAGAGGCTGATCTACGACACCTCTAAGCTGGCAAGCGGAGTGCCCTCCCGCTTCTCTGGCAGCGGCTCCGGCACCGACTTTACCCTGACAATCAACTCCCTGGAGGCCGAGGATGCCGCCACATACTATTGTCAGCAGTGGTCCTCTAATCCTTTCACCTTTGGCCAGGGCACAAAGGTGGAGATCAAGCGGCTCGAGCCCAAGAGCTGCGACAAGACCCACACCTGTCCTCCATGTCCTGCTCCAGAGTTTCAAGGCGGCCCTTCCGTGTTCCTGTTTCCTCCAAAGCCTAAGGACACCCTGTACATCACCCGCGAGCCTGAAGTGACCTGTGTGGTGGTGGATGTGTCCCACGAGGACCCCGAAGTGAAGTTCAATTGGTACGTGGACGGCGTGGAAGTGCACAACGCCAAGACCAAGCCTAGAGAGGAACAGTACAACAGCACCTACAGAGTGGTGTCCGTGCTGACCGTGCTGCACCAGGATTGGCTGAACGGCAAAGAGTACAAGTGCCAGGTGTCCAACAAGGCCCTGCCTGCTCCTATCGAGAAAACCATCAGCAAGGCCAAGGGCCAGCCTAGGGAACCTCAAGTGTACGTGTACCCTCCTAGCCGGGACGAGCTGACCAAGAATCAGGTGTCCCTGACCTGCCTCGTGAAGGGCTTCTACCCTTCCGACATCGCCGTGGAATGGGAGAGCAATGGCCAGCCTGAGAACAACTACAAGACAACCCCTCCTGTGCTGGACAGCGACGGCTCTTTTGCCCTGGTGTCCAAGCTGACAGTGGACAAGTCCAGATGGCAGCAGGGCAACGTGTTCAGCTGCAGCGTGATGCACGAGGCCCTGCACAACC ACTACACCCAGAAGTCCCTGAGCCTGTCTCCTGGATGA

CD45-IgG-hole

ATGGGCTGGAGCTGCATTATCCTGTTCCTGGTGGCCACAGCCACCGGCGTGCACAGCGAGGTGCAGCTGGTTGAATCTGGCGGAGGACTGGTTCAGCCTGGCGGATCTCTGAGACTGTCTTGTGCCGCCAGCGGCTTCACCTTCAACAACTACTGGATGACCTGGGTCCGACAGGCCCCTGGCAAAGGACTTGAATGGGTCGCCAGCATCTCTAGCAGCGGCGGCAGCATCTACTACCCCGATTCTGTGAAGGGCAGATTCACCATCAGCCGGGACAACAGCAAGAACACCCTGTACCTGCAGATGAACAGCCTGAGAGCCGAGGACACCGCCGTGTACTACTGTGCCAGAGATGAGAGATGGGCTGGCGCCATGGATGCTTGGGGACAGGGAACAACCGTGACCGTTTCTTCTGGCGGCGGAGGAAGCGGAGGCGGAGGCTCCGGTGGTGGTGGATCTGACATCCAGATGACACAGAGCCCCAGCAGCCTGTCTGCCTCTGTGGGAGACAGAGTGACCATCACATGCAAGGCCAGCCAGAACATCAACAAGAACCTGGATTGGTATCAGCAGAAGCCCGGCAAGGCCCCTAAGCTGCTGATCTACGAGACAAACAACCTGCAGACCGGCGTGCCCAGCAGATTTTCTGGCTCTGGCAGCGGCACCGACTTCACCCTGACCATATCTAGCCTGCAGCCTGAGGACTTCGCCACCTACTACTGCTACCAGCACAACAGCCGGTTCACCTTTGGCGGAGGCACCAAGCTGGAAATCAAGCGGCTCGAGCCCAAGAGCTGCGACAAGACCCACACCTGTCCTCCATGTCCTGCTCCAGAGTTTCAAGGCGGCCCTTCCGTGTTCCTGTTTCCTCCAAAGCCTAAGGACACCCTGTACATCACCCGCGAGCCTGAAGTGACCTGTGTGGTGGTGGATGTGTCCCACGAGGACCCCGAAGTGAAGTTCAATTGGTACGTGGACGGCGTGGAAGTGCACAACGCCAAGACCAAGCCTAGAGAGGAACAGTACAACAGCACCTACAGAGTGGTGTCCGTGCTGACCGTGCTGCACCAGGATTGGCTGAACGGCAAAGAGTACAAGTGCCAGGTGTCCAACAAGGCCCTGCCTGCTCCTATCGAGAAAACCATCAGCAAGGCCAAGGGCCAGCCTAGGGAACCTCAAGTGTACGTGTACCCTCCTAGCCGGGACGAGCTGACCAAGAATCAGGTGTCCCTGACCTGCCTCGTGAAGGGCTTCTACCCTTCCGACATCGCCGTGGAATGGGAGAGCAATGGCCAGCCTGAGAACAACTACAAGACAACCCCTCCTGTGCTGGACAGCGACGGCTCTTTTGCCCTGGTGTCCAAGCTGACAGTGGACAAGTCCAGATGGCAGCAGGGCAACGTGTTCAGCTGCAGCGTGATGCACGAGGCCCTGCACAACCACTA CACCCAGAAGTCCCTGAGCCTGTCTCCTGGATGA

SLAMF6-IgG-knob

ATGGGCTGGAGCTGCATTATCCTGTTCCTGGTGGCCACAGCCACCGGCGTGCACAGCCAGGTTCAGCTGGTGCAGTCTGGCAGCGAGCTGAAAAAACCTGGCGCCTCCGTGAAGGTGTCCTGCAAGGCTAGCGGCTACACCTTTACCAACTTCGGCATGAACTGGGTCCGACAGGCCCCTGGACAAGGCTTGGAATGGATGGGCTGGATCAACACCTACAGCGGCGAGGCCACATACGCCGACGATTTCAAGGGCAGATTCGTGTTCAGCCTGGACACCAGCGTGTCCACAGCCTACCTGCAGATCAGCTCTCTGAAGGCCGAGGACACCGCCGTGTACTACTGTGCTAGAAGAGGCGGCACCGCCGAGTTCGATTATTGGGGACAGGGCACCCTGGTCACCGTTTCTAGCGGAGGCGGAGGATCTGGTGGCGGAGGAAGTGGCGGAGGCGGTTCTGAAATTGTGCTGACACAGAGCCCCGACTTCCAGAGCGTGACCCCTAAAGAAAAAGTGACCATCACCTGTAGCGCCAGCAGCTCTATCAGCAGCAACTTCCTGCACTGGTATCAGCAGAAGCCCGATCAGAGCCCCAAGCTGCTGATCTACAGAACCAGCAAGCTGGCCTCTGGCGTGCCCAGCAGATTTTCTGGCTCTGGCTCCGGCACCGACTTCACCCTGACAATCAATAGCCTGGAAGCCGAGGATGCCGCCACCTACTATTGTCAGCAGGGCATCTACATGCCCCTGACCTTTGGCGGCGGAACAAAGCTGGAAATCAAGCGGCTCGAGCCCAAGAGCTGCGACAAGACCCACACCTGTCCTCCATGTCCTGCTCCAGAGTTTCAAGGCGGCCCTTCCGTGTTCCTGTTTCCTCCAAAGCCTAAGGACACCCTGTACATCACCCGCGAGCCTGAAGTGACCTGTGTGGTGGTGGATGTGTCCCACGAGGACCCCGAAGTGAAGTTCAATTGGTACGTGGACGGCGTGGAAGTGCACAACGCCAAGACCAAGCCTAGAGAGGAACAGTACAACAGCACCTACAGAGTGGTGTCCGTGCTGACCGTGCTGCACCAGGATTGGCTGAACGGCAAAGAGTACAAGTGCCAGGTGTCCAACAAGGCCCTGCCTGCTCCTATCGAGAAAACCATCAGCAAGGCCAAGGGCCAGCCTAGGGAACCTCAGGTGTACGTGTTGCCTCCTAGCAGGGACGAGCTGACCAAGAATCAGGTGTCCCTGCTGTGCCTGGTCAAGGGCTTCTACCCTTCCGACATCGCCGTGGAATGGGAGAGCAATGGCCAGCCTGAGAACAACTACCTGACCTGGCCTCCTGTGCTGGATAGCGACGGCTCATTCTTCCTGTACAGCAAGCTGACAGTGGACAAGAGCAGATGGCAGCAGGGCAACGTGTTCAGCTGCAGCGTGATGCACGAGGCCCTGCACAACCACTA CACCCAGAAGTCCCTGAGCCTGTCTCCTGGATGA
